# Assessment of Osstell ISQ’s reliability for implant stability measurement: A cross-sectional clinical study

**DOI:** 10.4317/medoral.19120

**Published:** 2013-10-13

**Authors:** Mariano Herrero-Climent, Rocio Santos-García, Reyes Jaramillo-Santos, Manuel M. Romero-Ruiz, Ana Fernández-Palacin, Pedro Lázaro-Calvo, Pedro Bullón, Jose V. Ríos-Santos

**Affiliations:** 1 Lecturer at the Master’s Degree in Periodontology and Implants at the University of Seville; 2 Full-time lecturer at the Department of Dentistry at the University of Seville; 3 Full Professor at the Department of Dentistry at the University of Seville

## Abstract

Resonance frequency analysis (RFA) allows assess implant stability by measuring implant oscillation frequency on the bone. RFA is an objective and non-invasive method for implant stability measurement, although scarce evidence has been provided so far on its reliability. Objectives: Assess the Osstell ISQ system’s reliability (i.e., its measurement reproducibility and repeatability) by means of the intraclass correlation coefficient (ICC) as statistical method. Study Desing: Implants stability registers were completed by means of Osstell ISQ on 85 implants on 23 patients. Six measurements were completed on each implant by means of two different SmartPegs (types I and II); that is, three consecutive measurements with each transducer. Results: Average ISQ was 72.40, 72.22 and 72.79, and 72.06, 72.59 and 72.82 in the first, second, and third measurements with SmartPegs I and II, respectively. Equal values or differences below three ISQ points were observed in 52.9% and 62.4% of the cases with SmartPegs I and II, respectively. The intraclass correlation coefficient was 0.97 for both SmartPegs, and repeatability and reproducibility also reached 0.97 for both SmartPegs. Conclusions: The RFA system Osstell ISQ presents almost perfect repeatability and reproducibility after intraclass correlation coefficient analysis. Osstell ISQ measurements are highly reliable regarding reproducibility. Therefore, one measurement proves enough.

** Key words:**Dental implants, RFA, ISQ, implant stability, Osstell.

## Introduction

Immediate load in implants has proven a predictable treatment, with similar survival and success rates to those obtained with traditional load (1-2). Immediate load’s success is closely related to the achievement of primary stability upon implant insertion, and the absence of micro-movements during the healing period (3-4). Primary implant stability upon placement depends on factors such as bone density, surgical technique employed and implant design (5-6). However, literature seems to point out that, among the involved factors, primary implant stability is the main factor for successful immediate loading, since it limits micro-movements in the bone-implant interface, thus favouring osseointegration (7-9). Thus, most studies conclude that poor stability is an exclusion criterion for immediate loading (9).

The implant osseointegration concept was defined as “direct, structural and functional contact between the living bone and a function ally loaded endo-osseous implant’s surface”(10). Clinical testing is currently available —although bone-implant contact cannot be measured, implant stability and, therefore, also implant osseointegration can be assessed and quantified (11).

Implant initial stability after placement has commonly been assessed according to mobility and bone quality following the Lekholm & Zarb jaw quality scale (12). In 1994, Johansson & Strid described a more objective method based on measuring shear resistance during milling. Other subjective methods such as implant percussion have also been used, yet with rather unpromising results. The removal torque is also a research method to assess implant stability, yet it is an invasive method which involves bone-implant interface failure and, therefore, clinically uninteresting (13-14).

The Periotest system designed by Schulte et al. (1983) to measure tooth mobility (15) was used by authors such as Teerlinck (16) as a quantitative method to assess implant stability. Olivé & Aparicio (1990) (17) reached success with this system, although this technique has been suggested to be sensitive to a wide range of factors such as tip placement angle, pillar height and metallic tip-implant distance, (18) as well as scarcely sensitive to differentiate between osseointegrated and non-osseointegrated implants. The instrument scale ranges from -8 to +50 Periotest values, and successful implants range from -5 to +5. (5,19).

Meredith et al. (20) described a noninvasive clinical method: the resonance frequency analysis (Osstell method). Osstell devices have been designed since 1999 by the Integration Diagnostics Ltd. Company (Sävedalen, Sweden). Within the last decade, several generations of this device have followed one another for implant stability measurement: Osstell, Osstell Mentor and Osstell ISQ. It is a noninvasive diagnosis technique that uses a piezoelectric transducer, which emits a sinusoidal signal within a specific frequency meant to make the implant vibrate. Implant resistance to vibration is measured by the device and transformed into an ISQ value (implant stability quotient -within a 0-100 scale, 100 being maximum implant stability). In 2009, the last generation of this device was developed: Osstell ISQ, which includes a new control unit with a probe connected to it by means of a cable.

Although its clinical use is progressively extending, literature review shows the absence of studies on the reproducibility and repeatability of its measurements for the RFA register and, therefore, on its dental implant stability measurement.

## Material and Methods

-Design of the Study: the present cross-sectional study is aimed at assessing system Osstell ISQ’s reliability (i.e., its measurement reproducibility and repeatability), thus assessing its clinical effectiveness upon dental implant stability measurement. It was developed in one only centre within the Master’s Degree in Periodontics and Implants at the University of Seville. The protocol of study, as well as the informed consent form, had been approved by the University of Seville’s Experimentation Ethics Committee.

-Patients: the RFA registers from patients who came to the university teaching hospital from September to December 2009, taken by means of the Osstell ISQ device, were analysed. Stability measurements were taken on a total number of 85 consecutive implants placed in 23 partially or completely toothless patients that were being restored with shot-blasted, rough surface dental implants from the Essential Cone®, Klockner Implant System. Patients were selected to meet the following inclusion criteria:

- Legal-age patients being treated from tooth absences by means of dental implants

- Collaborative patients

- Teeth to be replaced had to be extracted at least 4 months prior to implant insertion, with completely-healed bone crest

- Patient-signed informed consent form.

-Implants: measurements were taken on Essential Cone® implants, Klockner Implant System (three available diameters: 3.5, 4.0 and 4.5 mm-all of them with 4.5-mm platform-, and three available lengths: 8, 10 and 12 mm; chose according to bone availability in each case). All implants were placed within the Master’s Degree in Periodontics and Implants at the University of Seville in the aforementioned period. Surgery was completed by an experienced surgeon with at least 10 years of surgical experience and deep knowledge of the Klockner Implant System.

-Resonance Frequency Analysis (RFA)

The present study is aimed at assessing reliability in Osstell ISQ through the study of repeatability or evaluating the data obtained by one only transducer on the same implant, and reproducibility or evaluating the data obtained by several transducer on one only implant at the same time.

With this purpose two SmartPeg transducers were used on each implant, and three measurements were completed: therefore, total number of registers was six for each implant. All assessment was carried out consecutively regardless register time or location. Measurements were completed by one only experienced dentist with knowledge of the Osstell ISQ system for RFA assessment.

Both surgery (post-surgery registration) and control appointments were used to complete measurements, since the latter were independent from the degree of stability to be found. For registration the transducer or SmartPeg was directly screwed to the implant with the interposition of no prosthesis pillar. Manufacturer’s suggestions, summarised next, were followed:

- Use of SmartPeg or specific transducer for the 4.5-mm platform, Klockner Essential Cone System, for direct implant assessment

- No soft tissue interposition

- Manual transducer tightening 5-8 Ncm by a specific plastic screw-driver

- No contact between any part of the transducer and adjacent teeth

- Placement of the Osstell ISQ’s probe approximately 2 mm from the SmartPeg in a 90º angle relative to the implant’s major axis. In all cases the probe was oriented vestibular.

-Statistical analysis: data were statistically explored and debugged by means of numeric and graphic methods. To study concordance between consecutive measurements by the same device on the same patients, intraclass correlation coefficients (ICC) were calculated (confidence intervals set at 95%), and the hypotheses of null coefficients in the sampled population were assayed. We think that the most appropriate model to study ICC is that two-ways, mixed effects. To study reliability, ICC (21) were calculated (confidence intervals set at 95%), and the hypotheses of null coefficients in the sampled population were assayed.

To compare the values of paired numeric variables, Student’s T-test was applied to two related samples or Friedman’s test for comparison with more than 2 samples. Data analysis was completed with software package SPSS 17.0 for MS Windows (SPSS, Chicago, IL).

## Results

A total number of 85 implants in 23 patients were registered, out of which 81 implants were in posterior sections (36 maxilla/45 jaw). The remaining 4 implants were placed in the anterior maxillary section. Regarding implant length, 42 implants were 8-mm, 29 were 10-mm, and 14 were 12-mm long.

-Descriptive statistics

All measurements in the present study were considered valid; no registers were excluded. The overall average values obtained for the different groups ranged from 72.06 (Smartpegs I) and 72.82 ISQ (Smartpegs II).

**Figure 1:**
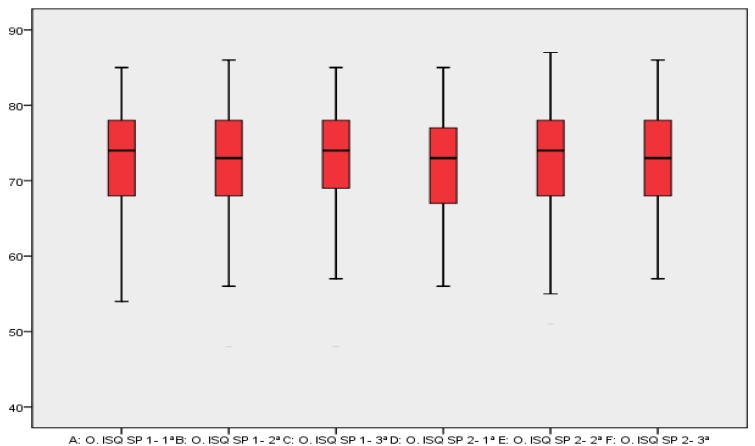
Correlation among the different register groups.

The average register obtained with SmartPeg I in its first measurement was 72.40 ISQ ± DT 7.012, while for the second and third measurements it was 72.22 ISQ ± DT 7.318, and 72.79 ISQ ± DT 7.208, respectively. On the other hand, the average register obtained with SmartPeg II in its first measurement was 72.06 ISQ ± DT 7.070, while for the second and third measurements it was 72.59 ISQ ± DT 7.404, and 72.82 ISQ ± DT 7.010, respectively. These values are reflected in figure 1, which shows appropriate correlation among the groups of registers. Regarding reliability analysis, the intraclass correlation coefficient (ICC) was 0.97 for both SmartPegs I and II, confidence interval at 95% (0.96-0.98), thus indicating an almost-perfect degree of concordance ( Table 1).

**Table 1:**
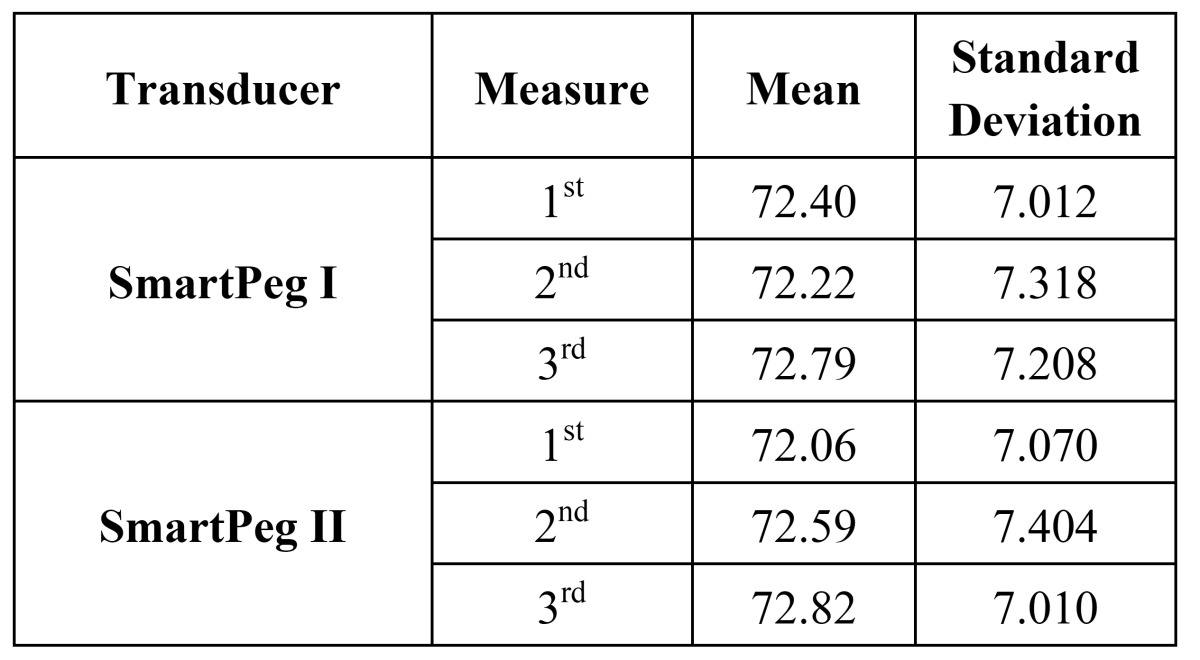
Mean values and standard deviation of ISQ values with both transducers.

-Analysis of value differences among registers: Differences among SmartPeg I measurements were analysed and classified according to differences: 0 = same value, 1-3 = difference of 1-3 ISQ points, 4-5 difference of 4-5 ISQ points, >5 = over-5-ISQ-point difference ( Table 2).

**Table 2:**
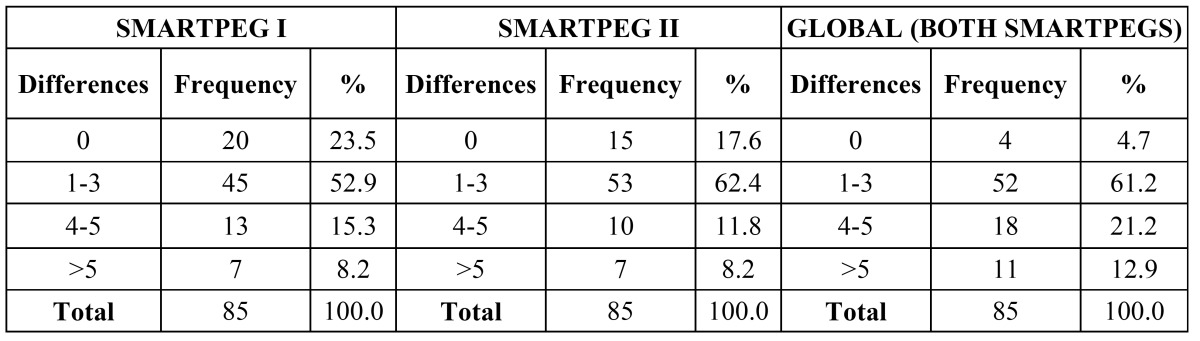
Measurement differences between SmartPegs I, II and global (all six measurements with both SmartPegs).

Thus, 76.5% of the registers of the first transducer and 80% of the register of the second transducer differed in 3 or less ISQ points. Subsequently, Smart Peg I and II measurements were compared to assess differences among the six completed measurements.

To evaluate concordance among the difference measurements completed with both SmartPegs, the intraclass correlation coefficient —based on an analysis of variance model with repeated or intrasubject measures— was used. Repeatability was 0.97 for both SmartPegs, while Osstell ISQ reproducibility was also 0.97.

## Discussion

Resonance Frequency Analysis (RFA) is a noninvasive intraoral method designed to assess bone-implant interface and may therefore provide clinical evidence of implant stability (20). Due to its high reproducibility and soundness, this technique has progressively, in the last years, outperformed the all techniques previously proposed to monitor implant stability (22).

Since 1996, numerous works have proven the RFA analysis system useful to obtain an objective assessment of implant stability. (23-24). RFA allows implant monitoring through sequential stability measurements, as well as indirect assessment of the influence of osseous remodelling around the implant on secondary implant stability.

However, there is scarce evidence of Osstell ISQ’s reliability upon measurement. Reliability is measured by means of concepts such as repeatability (i.e., several attempts with the same transducer lead to similar results), and reproducibility (i.e., different transducers on the same implant provide similar data). The lack of data on Osstell reliability was meant to be overcome by completing several registers and calculating the mean value. Hence, it is important the relying on a reliability study that contributes precise data on the device’s limits.

In the present study, to measure concordance between two quantitative assessments obtained with the same or different transducers, the intraclass correlation coefficient (equivalent to Cohen’s Kappa index) was calculated. It has been recommended to quantify reliability in clinical measurements, either by repeating measurements with the same device, or determining concordance between different instruments or observers under the same conditions. Value 1 has been considered equal to absolute concordance and the results of the present study was 0.97, Osstell ISQ’s repeatability and reproducibility can be inferred “almost perfect”, so multiple measurements are no longer necessary to assess the device’s reliability.

Meredith (1996) (26) studied the first generation of the Osstell system, and found high repeatability, but referring that though the transducer’s tightening torque was the only torque variable that can distort registers. The present study on the last generation of the Osstell system (Osstell ISQ) reports higher repeatability and reproducibility than Meredith’s, with no differences between them.

Brouwers et al. (25) completed a study on 32 implants placed on desiccated human jawbones to determine RFA system’s intra- and inter-observer reliability, and reported average-to-good values, according to the intraclass correlation index, for both intra-and inter-observer reliability. An implant removal torque after implant measurement was applied, and they found no correlation between RFA and removal torque values. Regarding the assessment of the RFA system’s intra-and inter-observer reliability our results can be considered almost perfect vs. good according to the Brouwers study. The Osstell ISQ device reached 0.97 in the intertransduce ICC assessment.

Lachmann et al. (2006) (22) compared Osstell and Periotest reliability on cow rib, registering three measurements without withdrawing the transducer, three additional measurements after transducer withdrawal and manual tightening, and three more after withdrawing and mechanical 10-N tightening. Once data were analysed with the ICC, the obtained results were similar to those contributed in the present study (i.e., “almost perfect”), highlighting that the system’s error range can have no clinical relevance. Similar results were reported by Zix et al. (2008) (26) Our experience (27-28) tells us that resonance frequency analysis is a highly useful method to assess primary stability and implant stability evolution, and should therefore become part of implantology daily routine.

## Conclusions

The results of the present study imply that the resonance frequency analysis system Osstell ISQ presents “almost perfect” reproducibility and repeatability after statistical analysis by means of the intraclass correlation coefficient. It can be therefore concluded that Osstell system measurements are highly reliable regarding repeatability. Therefore one only measurement could be sufficient.
